# Advanced glycation end products exacerbate lipopolysaccharide-induced acute lung injury with diabetes by promoting ferroptosis via AMP-activated protein kinase/acetyl-CoA carboxylase signaling

**DOI:** 10.1038/s41598-025-26647-0

**Published:** 2025-12-13

**Authors:** Yaqin Sun, Yipan Fan, Aiyun Xu, Chao Chen, Yuyu Lu, Qian Li, Weixing Ge

**Affiliations:** 1 Department of Critical Medicine, The Affiliated Jiangning Hospital of Nanjing Medical University, Nanjing, Jiangsu China; 2 Department of Emergency, Women’s Hospital of Nanjing Medical University, Nanjing Women and Children’s Healthcare Hospital, Nanjing Medical University, Nanjing, China; 3 Department of Endocrinology, The Affiliated Jiangning Hospital of Nanjing Medical University, Nanjing, Jiangsu China; 4 Department of Endocrinology, Nanjing First Hospital, Nanjing Medical University, Nanjing, Jiangsu China

**Keywords:** Advanced glycation end products, Ferroptosis, Acute lung injury, Acetyl-CoA carboxylase, Diabetes complications, Medical research

## Abstract

Diabetes increases susceptibility to acute lung injury (ALI), yet the mechanisms linking hyperglycemia to pulmonary damage remain incompletely understood. Here, we demonstrate advanced glycation end products (AGEs)—metabolic byproducts elevated in diabetes—as promoters of ferroptosis that contribute to ALI pathogenesis. Clinical analysis of 170 patients with sepsis-related ALI showed that diabetic individuals had heightened inflammation and reduced PaO₂/FiO₂ ratios. Bioinformatic analysis revealed overlapping ferroptosis-related gene signatures between DM and ALI. In lipopolysaccharide (LPS)-induced ALI mice with diabetes mellitus (DM), reducing AGEs levels attenuated inflammatory cell infiltration and pro-inflammatory cytokine production, decreased Fe²⁺ accumulation and malondialdehyde (MDA) levels, and increased the expression of glutathione peroxidase 4 (GPX4) and solute carrier family 7 member 11 (SLC7A11). In vitro experiments suggested that AGEs exacerbate ferroptotic injury in LPS-treated bronchial epithelial (BEAS-2B) cells partly by suppressing AMP-activated protein kinase (AMPK)/acetyl-CoA carboxylase (ACC) signaling, an effect mitigated by pharmacological AMPK activation. These findings support a potential mechanistic link between DM and ALI through AGEs-driven ferroptosis and raise the possibility that targeting the AMPK/ACC pathway could offer therapeutic benefit.

**Supplementary Information:**

The online version contains supplementary material available at 10.1038/s41598-025-26647-0.

## Introduction

ALI and its severe form, acute respiratory distress syndrome (ARDS), are life-threatening conditions caused by pneumonia and non-pulmonary pathogenic factors. Hallmarks include pulmonary edema, atelectasis, refractory hypoxemia, and pulmonary infiltration^1,2^. The pathogenesis of ALI involves increased endothelial permeability, alveolar epithelial injury and dysfunction, as well as dysregulated pulmonary inflammation^3^. A study of 459 patients from 50 countries has reported a high prevalence (10.4%) and mortality rate (34.9%−46.1%) of ARDS in intensive care units^4^. Despite advances in supportive care, effective pharmacological treatments remain elusive.

DM is an independent risk factor for ARDS in patients infected with COVID-19^5^. In COVID-19 patients, elevated fasting blood glucose (FBG) significantly increases ARDS risk^6^, yet the underlying mechanisms remain poorly defined. Ferroptosis, a regulated form of cell death driven by iron-dependent lipid peroxidation and excessive reactive oxygen species (ROS)^7^, has emerged as a contributor to ALI. Morphologically, ferroptotic cells exhibit a loss of plasma membrane integrity, cytoplasmic and organelle swelling, and mitochondrial abnormalities such as condensation or swelling, increased membrane density, and reduced or absent crista^8^. Recent studies have shown that inhibiting ferroptosis alleviates LPS-induced lung injury, highlighting its therapeutic relevance^9–11^. Moreover, ferroptosis is also implicated in diabetic complications such as nephropathy, cardiomyopathy, and encephalopathy^12–14^. However, whether hyperglycemia exacerbates ALI by promoting ferroptosis has not been determined.

AGEs are a group of compounds formed through a series of nonenzymatic reactions involving amino groups of proteins and carbonyl groups of reducing sugars, particularly under hyperglycemic or oxidative stress conditions^15^. Emerging evidence links AGEs to the induction of ferroptosis^12,16^, but their role in ferroptosis within alveolar epithelial cells has not been explored.

Here, we demonstrate that AGEs promote ferroptosis in LPS-induced ALI with DM and elucidate the regulatory role of AMPK/ACC signaling. Through an integrative approach combining clinical data, bioinformatics, and in vivo and in vitro models, we explore a potential mechanism by which DM exacerbates ALI through AGEs-mediated ferroptosis. These findings suggest a potential therapeutic axis for ALI/ARDS in diabetic individuals.

## Methods

### Patient grouping and clinical data collection

A total of 170 patients with sepsis-related ALI were recruited from the Affiliated Jiangning Hospital of Nanjing Medical University between January 2022 and January 2025. All patients met the diagnostic criteria for sepsis as defined by the 2016 International Guidelines for the Management of Sepsis and Septic Shock, and for ALI according to the 2006 guidelines for ALI/ARDS Diagnosis and Treatment. Patients were categorized into either the ALI + DM group (*n* = 60) or the ALI group (*n* = 110) based on the presence or absence of DM. Blood samples were collected within 24 h of admission, and the levels of C-reactive protein (CRP), procalcitonin (PCT), white blood cell (WBC), FBG, interleukin-6 (IL-6), as well as the PaO2/FiO2 ratio, were measured. This study involving human participants was conducted in accordance with the Declaration of Helsinki. The protocol was approved by the Ethics Committee of Jiangning Hospital (No. 2025-03-025-K01), and all patients in this study signed the letter of consent.

### Bioinformatics methods

Microarray datasets were obtained from the Gene Expression Omnibus (GEO) database (https://www.ncbi.nlm.nih.gov/geo/). The GSE95849 dataset contains RNA expression profiles of peripheral blood mononuclear cells from 6 patients with DM and 6 healthy individuals. The GSE10474 dataset contains RNA expression profiles of whole blood from 13 patients with ALI and sepsis and 21 patients with sepsis alone. Ferroptosis-related genes were downloaded from the FerrDb database (http://www.zhounan.org/ferrdb/current/operations/download.html). Differentially expressed genes (DEGs), along with the Kyoto Encyclopedia of Genes and Genomes (KEGG) pathway and Gene Ontology (GO) enrichment analyses, were conducted using R software. *P* < 0.05 and |log2FC| > 1 were defined as the screening criteria for DEGs. Overlapping DEGs and volcano plots were generated using the online tool (https://www.omicstudio.cn/tool).

### Animal model

Twenty, 8 weeks old, specific pathogen-free male C57BL/6J mice (18–20 g) were purchased from Nanjing Medical University (Nanjing, China) and raised under standard conditions. All animal procedures complied with the National Institutes of Health Laboratory Animal Care and Use Guidelines, and all methods are reported in accordance with ARRIVE guidelines. All mice were randomly divided into 2 groups: the Con group (*n* = 10) and the DM group (*n* = 10). Mice in the Con group were fed a normal chow diet while those in the DM group were fed a high-fat diet (HFD, 60% kcal from fat, 20% kcal from protein, and 20% kcal from carbohydrate, Research Diets, NK) for 4 weeks to induce insulin resistance before being intraperitoneally injected with 40 mg/kg/day streptozotocin (STZ, Yuanye, China) for 3 days to induce partial insulin deficiency and hyperglycemia, mice with FBG ≥ 11.1 mmol/L were considered to be a successful model of diabetes. Subsequently, the mice in the DM group were further randomly subdivided into 2 subgroups: the DM + ALI group (*n* = 5) and the DM + ALI + PYR group (*n* = 5). The mice in the Con group were further randomly subdivided into 2 subgroups: the Con group (*n* = 5) and the ALI group (*n* = 5). Mice in the DM + ALI + PYR group received an intragastric administration of 400 mg/kg/day AGEs inhibitor pyridoxamine (PYR, Sigma-Aldrich, USA) for 12 weeks, while the mice in other groups received an equal volume of normal saline. Subsequently, ALI was induced in the ALI, DM + ALI, and DM + ALI + PYR groups via intraperitoneal injection of 5 mg/kg LPS (Sigma, Escherichia coli 0111:B4) following anesthesia while the mice in the Con group were intraperitoneally injected with PBS instead. 12 h later, all mice were euthanized. Venous blood, bronchoalveolar lavage fluid (BALF), and lung tissues were collected. Venous blood and BALF were centrifuged to obtain serum and supernatant. Mice were anesthetized using sodium pentobarbital (50 mg/kg, intraperitoneally) and euthanized by cervical dislocation.

### Cell culture and treatment

BEAS-2 cells (Procell, Wuhan, China) were cultured in RPMI-1640 medium supplemented with 1% penicillin-streptomycin and 10% fetal bovine serum (FBS) at 37℃ in a 5% CO_2_ atmosphere. To evaluate the effects of LPS, AGEs (Bioss, China), AMPK activator AICAR (Selleck, USA), and ferroptosis inhibitor ferrostatin-1 (Fer-1, Sigma) on BEAS- 2B cell viability, cell viability was tested by CCK8 assay kit. To investigate whether AGEs promote ferroptosis via inhibition of AMPK activation, cells were treated with AICAR (1 mM) in the presence of LPS (5 µg/ml) and AGEs (100 µg/ml). Then cells were collected for subsequent analyses.

### Cell viability test

The CCK8 assay (Beyotime, China) was performed to assess cell viability. BEAS-2B cells were seeded into 96-well plates at a density of 5 × 10^3^ cells/well and cultured for 24 h. The medium was then replaced with fresh medium containing the indicated concentrations of LPS (0, 1, 5, and 10 µg/ml), AGEs (0, 10, 100, and 1000 µg/ml), AICAR (1 mM), and Fer-1 (2 µM). After incubation for the designated period, 10 µl CCK-8 reagent was added to each well, followed by incubation at 37℃ in the dark for 2 h. Measure the absorbance at wavelength 450 nm with an automatic biochemical analyzer (Infinite M200 Pro, Switzerland).

### Measurement of MDA and Fe^2+^ levels

MDA and Fe^2+^ levels in lung tissues and cell lysates were measured using the MDA Assay Kit (Beyotime, China) and the Ferrous Iron Colorimetric Assay Kit (Elabscience, China), respectively, according to the manufacturer’s instructions.

### Enzyme-linked immunosorbent assay

The levels of tumor necrosis factor-α (TNF-α, Cusabio, China), interleukin-1β (IL-1β, Cusabio, China), and interleukin-6 (IL-6, Cusabio, China) in BALF supernatants, as well as serum AGEs (Nanjing Jiancheng, China) levels, were measured using ELISA kits according to the manufacturer’s protocols.

### Haematoxylin–eosin (HE) staining

The lungs of mice were fixed in 4% paraformaldehyde for 48 h, then dehydrated with graded ethanol, embedded in paraffin, and sectioned at 3 μm thickness. Sections were dewaxed, hydrated, and stained with hematoxylin and eosin. Lung injury was assessed under an inverted fluorescent microscope (OLYMPU IX51, China). Assessment criteria of lung injury: pulmonary edema, neutrophil infiltration, alveolar and interstitial congestion, and hyaline membrane formation. A scoring system from 0 to 4 was used to describe the severity (0 = none, 1 = mild, 2 = moderate, 3 = severe, 4 = very severe).

### Immunohistochemistry

Lung sections were dewaxed, rehydrated, and blocked with 10% goat serum. They were then incubated overnight at 4 °C with primary antibodies (P-AMPK and P-ACC), followed by incubation with secondary antibodies at room temperature for 1 h. The catalog numbers and the working dilutions of antibodies were listed in Table S1. Finally, images were acquired by an inverted fluorescent microscope.

### Immunofluorescence staining

Cell samples were rinsed 3 times, fixed with 4% paraformaldehyde for 30 min, permeabilized with 0.1% Triton X-100 for 10 min, and blocked with 10% goat serum for 1 h. Cells were then incubated overnight with primary antibodies (P-AMPK and P-ACC) at 4℃. The next day, primary antibodies were discarded and the samples were rinsed once more before being incubated with fluorescent secondary antibodies (Invitrogen, USA) in the dark at room temperature for 1 h. Nuclei were counterstained with DAPI. Fluorescence images were captured using a fluorescence microscope.

### Western blot analysis

Cells and lung tissues were lysed in RIPA buffer (Beyotime, China) supplemented with protease and phosphatase inhibitor (Beyotime, China), and the protein concentrations were measured using the BCA assay kit (Beyotime, China). SDS-PAGE (EpiZyme, China) was used to separate the proteins, which were then electrotransferred onto PVDF membranes (Merck, Germany). After blocking with a Western blocking solution (Beyotime, China) at room temperature for 1 h, the membranes were incubated with primary antibodies overnight at 4℃. Before 1 h of incubation with secondary antibody at room temperature, the membranes were washed three times with TBST (Servicebio, China). Finally, protein blots were detected by the super-sensitive ECL chemiluminescence kit (Beyotime, China).

### Total RNA isolation and quantitative Real-Time polymerase chain reaction (PCR)

Total RNAs of BEAS-2B cells were extracted using the FastPure Cell/Tissue Total RNA Isolation Kit V2 (Vazyme, China). The cDNA was synthesized using 1 µg of the total RNA with the HiScript III RT SuperMix for qPCR (Vazyme, China). Then the real-time PCR was performed with ChamQ SYBR qPCR Master Mix (Vazyme, China) on a Step One Plus Real-Time PCR System (Applied Biosystems, USA). The sequences of different primers were listed in Table S2.

### Statistical analysis

GraphPad Prism 8 and SPSS Statistics 21 software were used for statistical analysis. Data are presented as mean ± standard deviation (SD) if normally distributed. For comparisons between two independent sample groups, the Student’s t-test was used, while for single-factor comparisons among three or more groups, we used a one-way analysis of variance (ANOVA). For non-normally distributed data, the rank sum test was used. *P* < 0.05 was considered statistically significant.

### Ethics approval

The clinical study was reviewed and approved by the Ethics Committee of Jiangning Hospital (No. 2025-03-025-K01). All patients in this study signed the letter of consent. All animal experiments were approved by the Animal Ethics Committee of Nanjing Medical University (NO: 2306039).

## Results

### Inflammation and oxygenation are more severely affected in sepsis-related ALI patients with DM

**Fig. 1:**
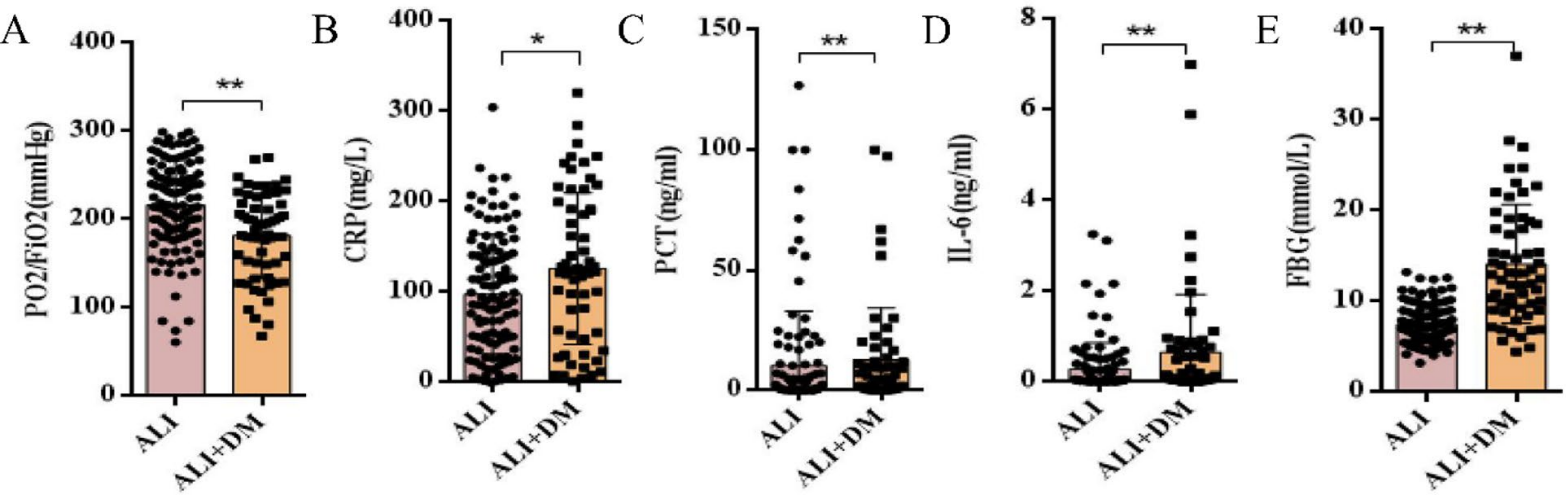
Comparison of clinical indicators between sepsis-related ALI patients with and without diabetes. (A) PaO₂/FiO₂ ratio, (B) CRP levels, (C) PCT levels, (D) IL-6 levels, and (E) FBG levels were compared between the two groups. Error bars represent SD, *P < 0.05, **P < 0.01, * versus ALI group.

Since DM has been reported to worsen systemic inflammation and impair lung function, we first examined whether DM aggravates inflammation and oxygenation impairment in patients with sepsis-related ALI. A total of 170 patients with sepsis-related ALI were enrolled and categorized into the ALI + DM group (*n* = 60) or the ALI group (*n* = 110) based on the presence or absence of DM. Compared to the ALI group, patients in the ALI + DM group exhibited significantly higher levels of CRP, PCT, IL-6, and FBG. Additionally, the PaO₂/FiO₂ ratio was significantly lower in the ALI + DM group, suggesting more severe respiratory impairment. No significant differences were observed in age or WBC counts between the two groups (Table 1; Fig. 1).

**Table 1:** Clinical data of patients with sepsis-related ALI with and without DM.

	Groups	Gender(Male/Female)	Rank mean	Sum of ranks	Mann-Whitney U	Wilcoxon W	Z	*P*
Age (yr)	ALI	89/21	83.2	9152	3047	9152	−0.825	0.409
	ALI + DM	37/23	89.72	5383				
PO2/FiO_2_ (mmHg)	ALI	89/21	97.42	10716.5	1988.5	3818.5	−4.277	0.001
	ALI + DM	37/23	63.64	3818.5				
WBC (10^9^/L)	ALI	89/21	81.1	8921	2816	8921	−1.578	0.114
	ALI + DM	37/23	93.57	5614				
CRP (mg/L)	ALI	89/21	79.6	8756	2651	8756	−2.116	0.034
	ALI + DM	37/23	96.32	5779				
PCT (ng/ml)	ALI	89/21	76.85	8454	2349	8454	−3.101	0.002
	ALI + DM	37/23	101.35	6081				
IL-6 (ng/ml)	ALI	89/21	76.88	8457	2352	8457	−3.107	0.002
	ALI + DM	37/23	101.3	6078				
FBG (mmol/L)	ALI	89/21	64.65	7111	1006	7111	−7.48	0.001
	ALI + DM	37/23	123.73	7424				

### The overlapping DEGs and signaling pathways in DM and ALI

DM is associated with chronic low-grade inflammation in the organs characterized by increased activation of innate immune cells and elevated cytokines levels^17^, and accumulating evidence supports a potential link between DM and ALI. To identify shared signaling pathways and DEGs between DM and ALI, we analyzed two gene expression datasets: GSE95849 and GSE10474.

**Fig. 2:**
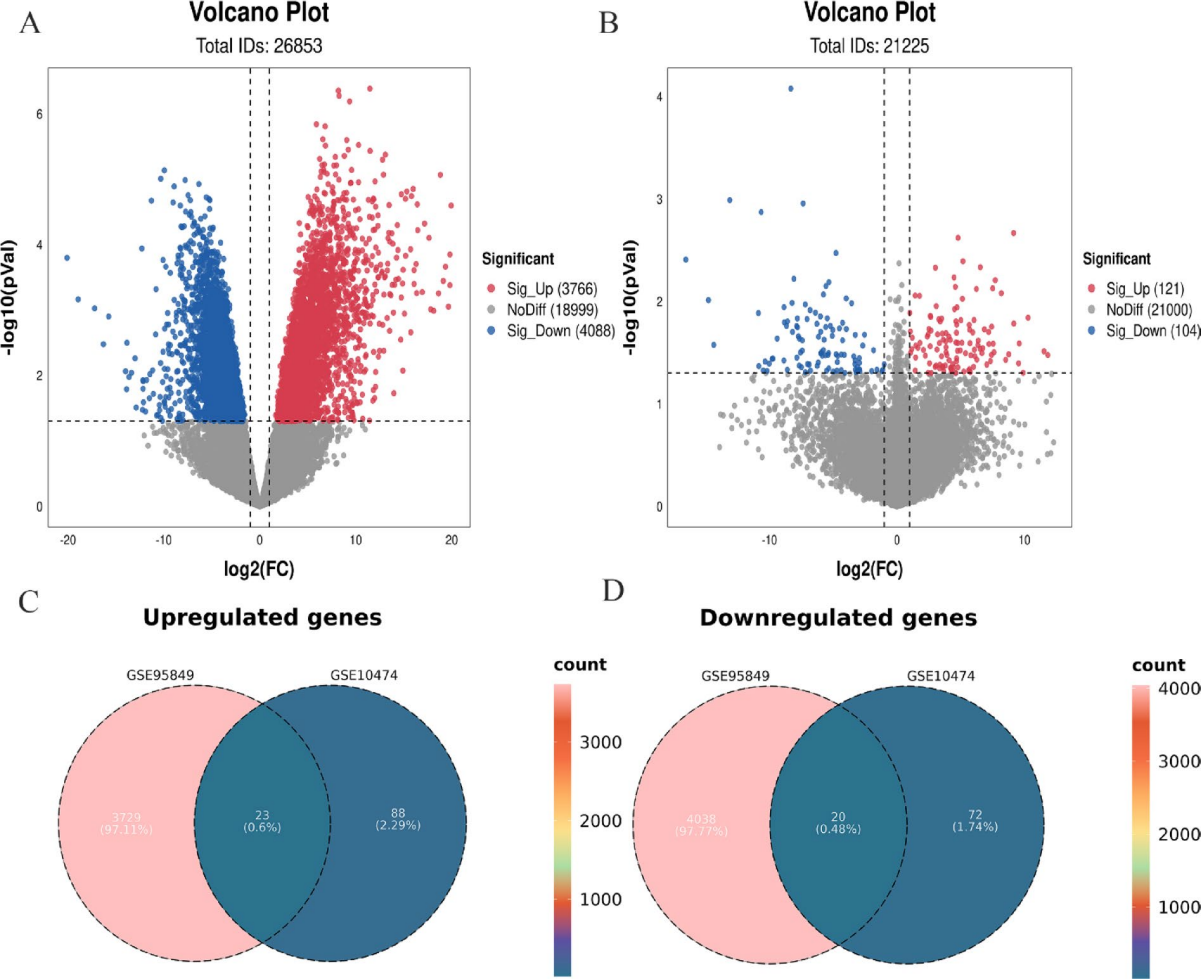
Volcano plots and Venn diagrams of DEGs from two gene expression datasets. (A–B) Volcano plots showing DEGs identified in each dataset. Red dots represent upregulated genes; blue dots represent downregulated genes; gray dots represent non-significant genes. DEGs were defined using the criteria: |log₂FC| > 1 and *P* < 0.05. (C–D) Venn diagrams showing overlapping upregulated and downregulated DEGs between the two datasets.

In dataset GSE95849, a total of 7,854 DEGs were identified from 26,853 detectable genes, with 3,766 upregulated and 4,088 downregulated. In GSE10474, 225 DEGs were identified out of 21,225 detectable genes, with 121 upregulated and 104 downregulated (Fig. 2A-B). After removing probe sets without corresponding gene symbols and averaging the expression of genes with multiple probe sets, we retained 3,752 upregulated and 4,058 downregulated DEGs in GSE95849, and 111 upregulated and 92 downregulated DEGs in GSE10474. By intersecting the DEGs from both datasets, we identified 23 overlapping upregulated and 20 overlapping downregulated DEGs by the Venn diagram (Fig. 2C-D).

**Fig. 3:**
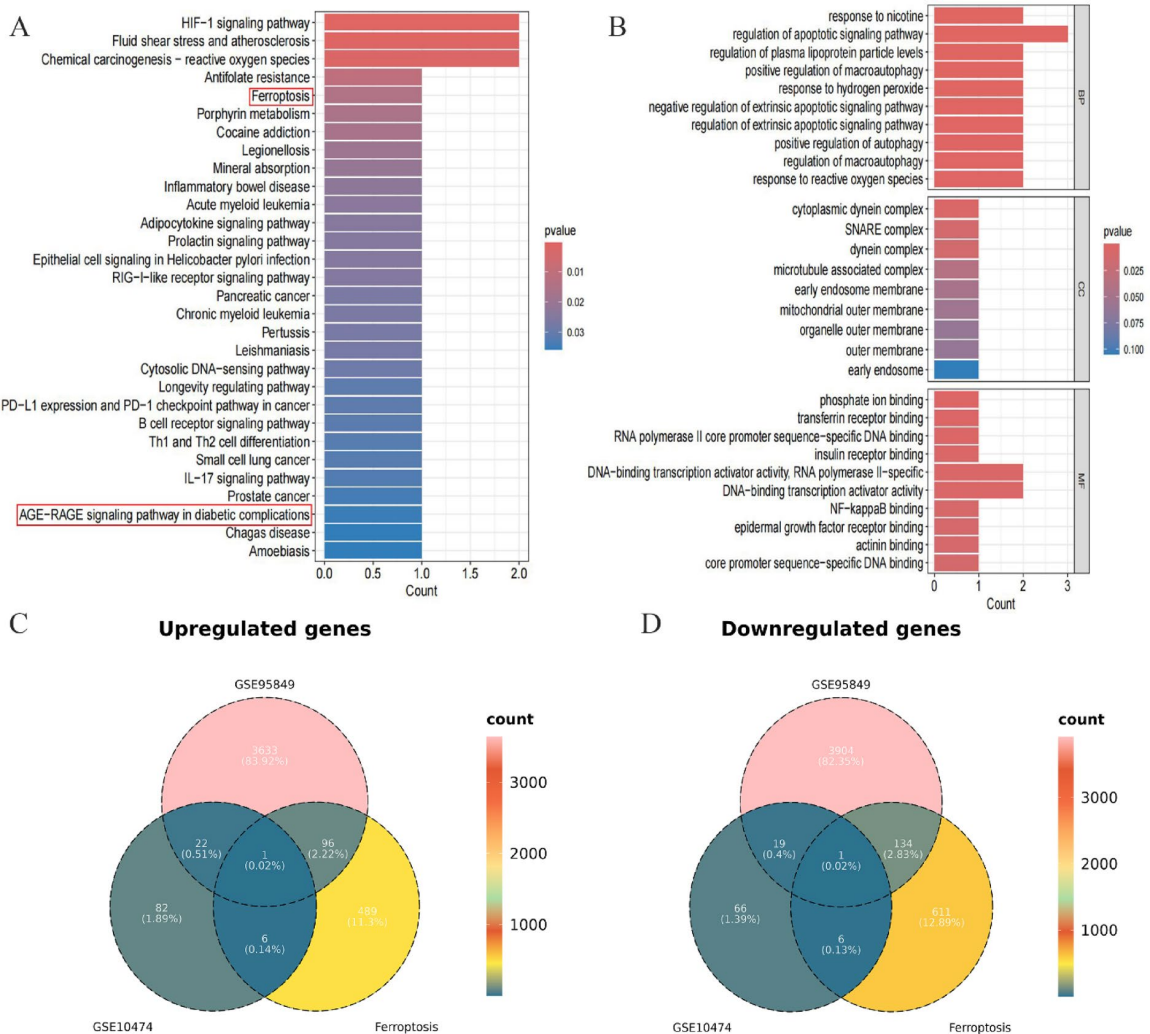
Functional enrichment and overlap analysis of DEGs and ferroptosis-related genes. (A) KEGG pathway enrichment analysis based on DEGs from the two gene expression datasets. Source: KEGG pathway database (Kanehisa Laboratories, Kyoto, Japan; citation guidelines: www.kegg.jp/kegg/kegg1.html). (B) GO enrichment analysis of DEGs from the two datasets. (C) Venn diagram showing overlapping upregulated DEGs with ferroptosis driver and marker genes. (D) Venn diagram showing overlapping downregulated DEGs with ferroptosis suppressor genes.

To explore the signaling pathways and biological functions of these overlapping DEGs, KEGG and GO enrichment analyses were performed. KEGG analysis revealed significant enrichment in pathways related to the ferroptosis and AGE − RAGE signaling pathway in diabetic complications (Fig. 3A). GO analysis showed that the overlapping DEGs were mainly involved in biological processes such as regulation of apoptotic signaling pathway, regulation of plasma lipoprotein particle levels and so on (Fig. 3B). Furthermore, Venn diagram analysis was used to identify the intersecting genes by calculating the overlap of the ferroptosis driver and marker genes with up-regulated DEGs from both datasets (Fig. 3C), and the ferroptosis suppressor genes with down-regulated DEGs from both datasets (Fig. 3D), respectively. Two intersecting genes were identified, which may indicate a potential involvement of ferroptosis in the pathophysiology of both DM and ALI.

### DM aggravates LPS-induced ALI via AGEs accumulation in vivo

**Fig. 4:**
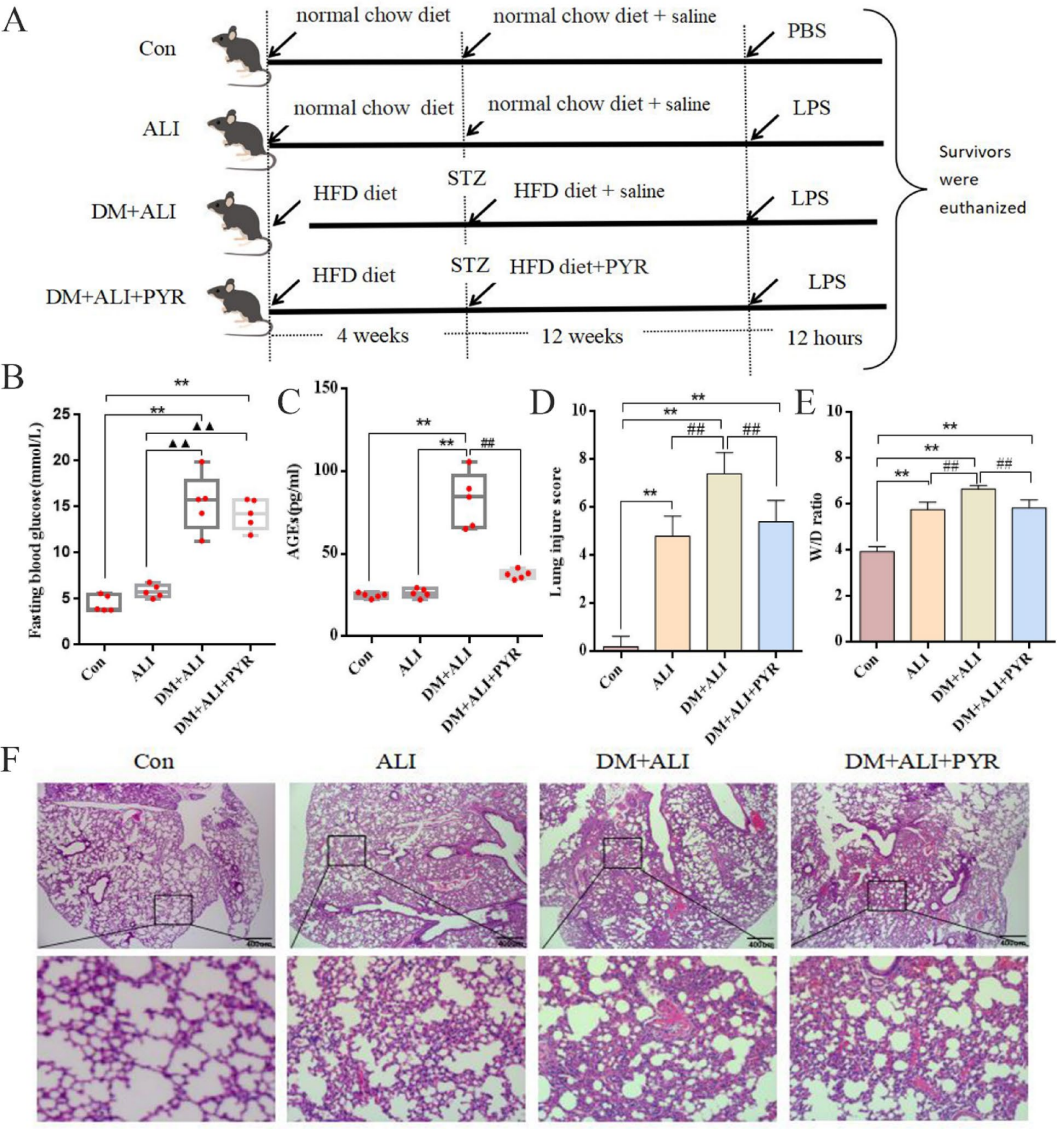
Diabetes aggravates LPS-induced ALI via AGEs accumulation in vivo. (A) Schematic diagram of mouse grouping and treatment protocols. (B) FBG levels. (C) Serum AGEs levels. (D) Lung injury scores. (E) Lung W/D ratio. (F) Representative HE-stained lung sections. *n* = 5; data are representative of three independent experiments. Scale bars: 400 μm. **P < 0.01, * versus Con group; ##*P* < 0.01, # versus DM + ALI group; ▲▲*P* < 0.01, ▲versus ALI group.

Since AGEs accumulate in diabetes and are implicated in ferroptosis^12^, we next investigated whether AGEs contribute to the exacerbation of LPS-induced ALI in diabetic mice. All mice were randomly divided into four groups: Control, ALI, DM + ALI, and DM + ALI + PYR (Fig. 4A). The results showed that mice in the DM + ALI and DM + ALI + PYR groups had a significantly higher FBG level than those in the Con and ALI groups (Fig. 4B). The AGEs levels in the DM + ALI group were higher compared to the Con, ALI, and DM + ALI + PYR groups (Fig. 4C). The above results suggest that pyridoxamine treatment was associated with reduced AGEs accumulation in diabetic mice.

Histological analysis of lung tissue using HE staining revealed varying degrees of lung injury among the groups. The lung injury score was highest in the DM + ALI group, followed by the DM + ALI + PYR and ALI groups, with minimal injury observed in the Con group (Fig. 4D). Pulmonary edema, assessed by the lung wet-to-dry (W/D) weight ratio, was significantly increased in the ALI group compared to the Con group and was further elevated in the DM + ALI group. Notably, treatment with pyridoxamine significantly reduced the W/D ratio in the DM + ALI + PYR group compared to the DM + ALI group (Fig. 4E). Morphological assessment of lung tissues further confirmed the severity of the injury. Mice in the DM + ALI group displayed severely destroyed pulmonary architecture, thickened alveolar septa, and notable inflammatory cell infiltration, while pyridoxamine partially alleviated these changes (Fig. 4F).

Collectively, these results indicate that DM exacerbates LPS-induced lung injury in mice, and lowering AGEs levels with pyridoxamine is associated with attenuated lung injury. These findings highlight a potential role of AGEs in the progression of LPS-induced ALI under diabetic conditions.

### DM aggravates ferroptosis and inflammation in LPS-induced ALI via AGEs accumulation in vivo

To determine whether AGEs contribute to inflammation and ferroptosis in diabetic ALI, we measured proinflammatory cytokines and immune cell infiltration in BALF and assessed ferroptosis-related biomarkers in lung tissues. Specifically, IL-6, IL-1β, TNF-α, WBCs, and neutrophils were used to evaluate inflammation, while Fe²⁺, MDA, GPX4, and SLC7A11 (also commonly known as xCT) served as ferroptosis indicators.

**Fig. 5:**
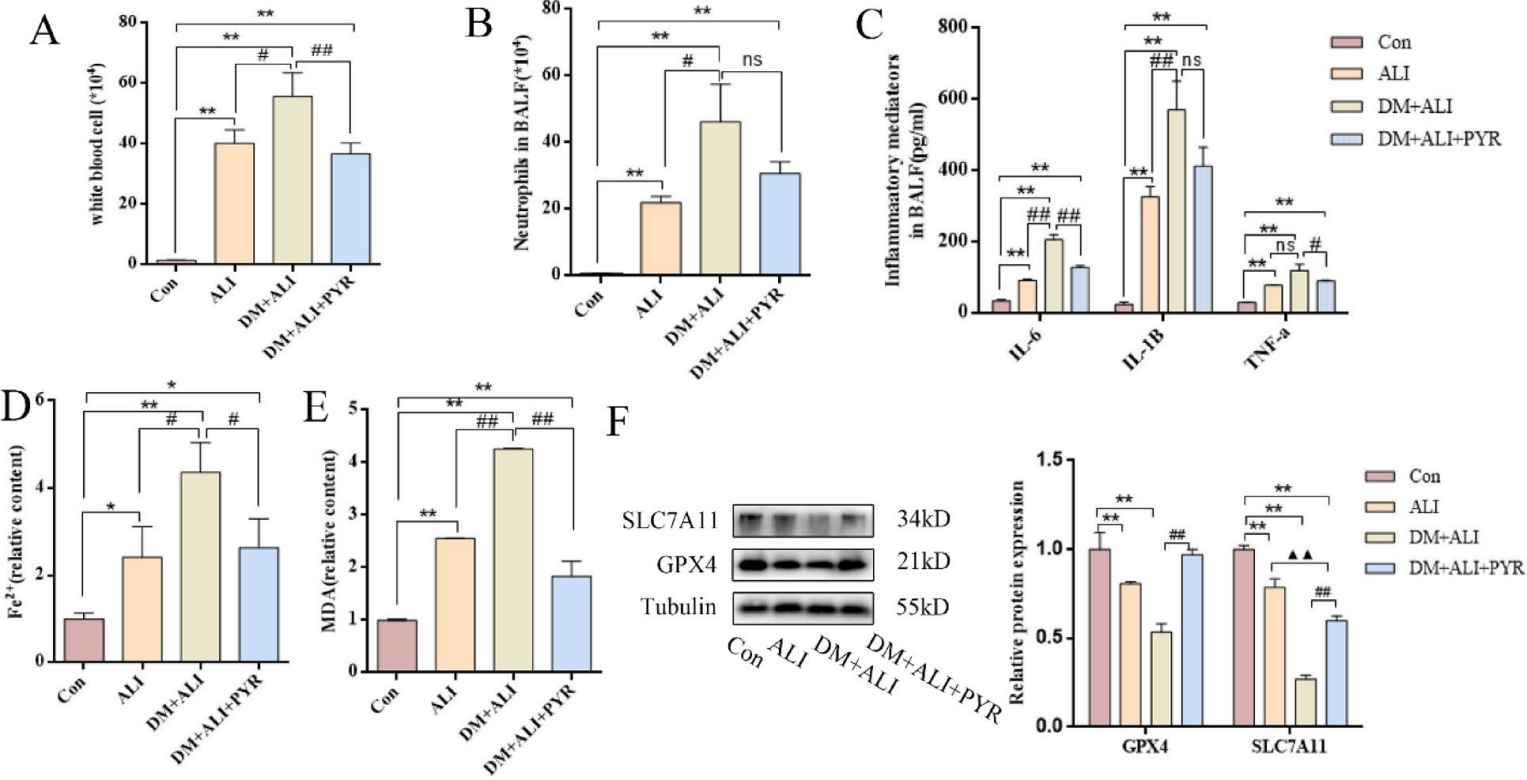
Diabetes aggravates ferroptosis and inflammation in LPS-induced ALI via AGEs accumulation in vivo. (A)WBC count in BALF. (B) Neutrophil count in BALF. (C) Levels of pro-inflammatory cytokines in BALF. (D) Fe²⁺ levels in lung tissues. (E) MDA levels in lung tissues. (F) Protein expression levels of GPX4 and SLC7A11 in lung tissues. *n* = 5, data are representative of three independent experiments. *P < 0.05, **P < 0.01, * versus Con group; #*P* < 0.05, ##*P* < 0.01, # versus DM + ALI group; ^▲▲^*P* < 0.01, ^▲^versus ALI group.

Compared to the Con group, levels of WBC, neutrophils, IL-6, IL-1β, and TNF-α in BALF were significantly increased in the ALI group, with even higher levels observed in the DM + ALI group. Notably, treatment with pyridoxamine in the DM + ALI + PYR group significantly reduced the levels of WBC, IL-6, and TNF-α compared to the DM + ALI group (Fig. 5A-C).

In terms of ferroptosis biomarkers, Fe^2+^ and MDA levels in lung tissues were elevated in the ALI group relative to the Con group, and further increased in the DM + ALI group. However, these levels were markedly reduced in the DM + ALI + PYR group compared to the DM + ALI group (Fig. 5D-E). Western blot analysis showed that compared to the Con group, GPX4 and SLC7A11 protein expression levels of mice lung tissues were decreased in the ALI group and further downregulated in the DM + ALI group. Pyridoxamine treatment significantly restored GPX4 and SLC7A11 protein expression in the DM + ALI + PYR group (Fig. 5F).

These findings suggest that the diabetic state, potentially via AGEs accumulation, contributes to ferroptosis and inflammation in LPS-induced ALI in vivo. Moreover, pyridoxamine treatment was associated with reduced inflammatory responses and ferroptosis, suggesting a potential role of AGEs in mediating these processes in diabetic ALI mice.

###  AGEs promote ferroptosis in the LPS-induced BEAS-2B cell injury model in vitro

**Fig. 6:**
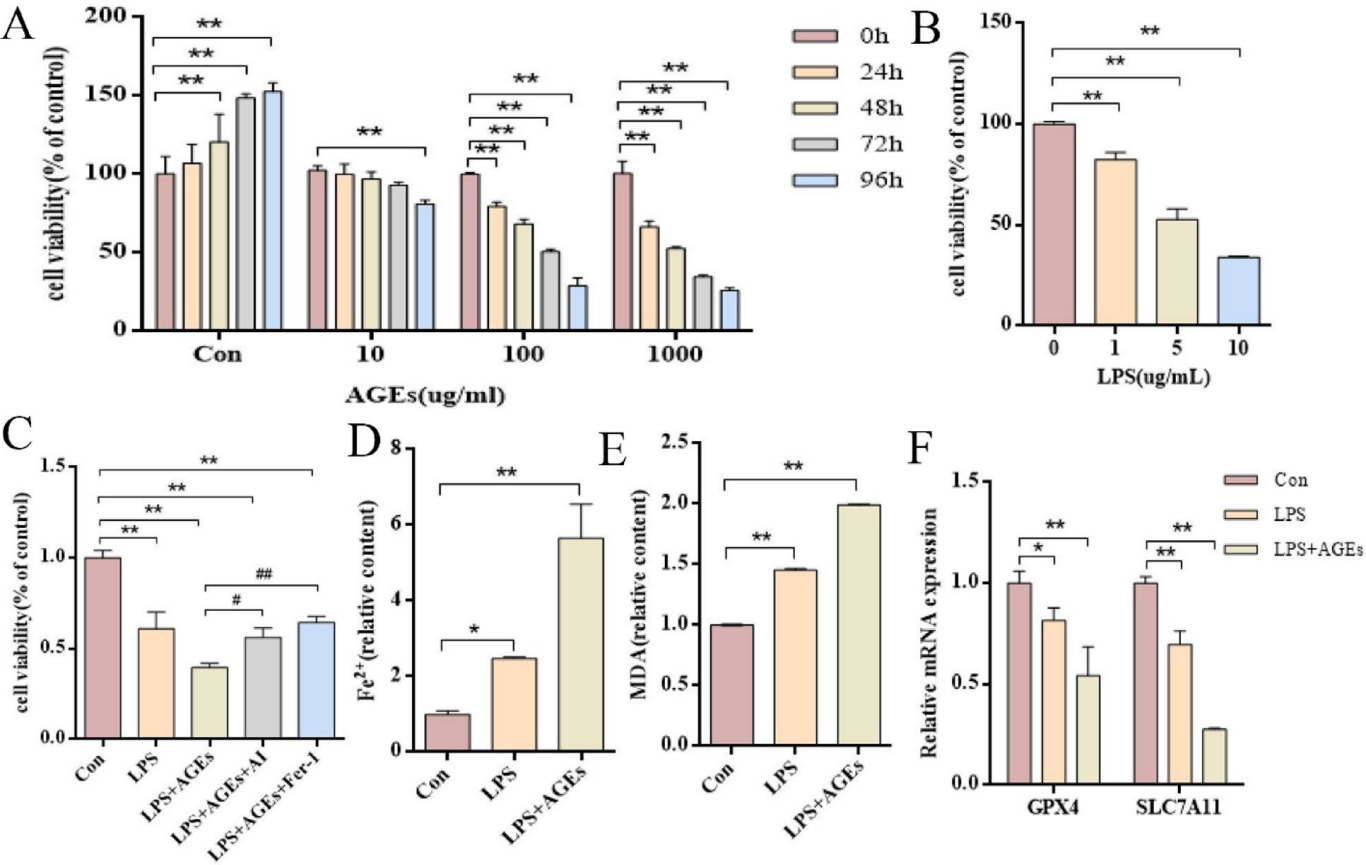
AGEs promote ferroptosis in the LPS-induced BEAS-2B cell injury model in vitro. (A) Cell viability of BEAS-2B cells treated with different concentrations of AGEs for varying periods. (B) Cell viability of BEAS-2B cells treated with different concentrations of LPS for 12 h. (C) Cell viability of BEAS-2B cells pretreated with 100 µg/ml AGEs, followed by co-treatment with 5 µg/mL LPS, 5 µg/mL LPS + 1 mM AICAR, 5 µg/mL LPS + 2 µM Fer-1, respectively. (D) Intracellular Fe²⁺ levels in the three experimental groups. (E) MDA levels in the three experimental groups. (F) Relative mRNA expression of SLC7A11 and GPX4 in the three experimental groups was determined by qPCR. Data are representative of three independent experiments, *P < 0.05, **P < 0.01, * versus Con group.

Given the association between AGEs and ferroptosis observed in diabetic ALI mice, we next examined their effects in lung epithelial cells. In BEAS-2B cells, AGEs reduced cell viability in a dose- and time-dependent manner (Fig. 6A). According to the viability results, we selected 100 µg/ml AGEs (72 h) and 5 µg/ml LPS (12 h) for subsequent experiments (Fig. 6A-B). Notably, co-treatment with the AMPK activator AICAR or the ferroptosis inhibitor Fer-1 significantly alleviated LPS + AGEs-induced cytotoxicity (Fig. 6C).

We next examined ferroptosis levels across treatment groups. Compared to the Con group, the LPS group exhibited increased levels of Fe²⁺ and MDA, along with down-regulated mRNA expression of GPX4 and SLC7A11 (Fig. 6D-F), indicating that LPS induced BEAS-2B cells ferroptosis. These effects were further enhanced in the LPS + AGEs group, suggesting that AGEs amplify LPS-induced ferroptosis in BEAS-2B cells.

###  AGEs regulate ferroptosis via AMPK-mediated phosphorylation of ACC

To investigate the mechanism by which AGEs exacerbate LPS-induced ferroptosis, we examined the AMPK/ACC signaling pathway. Since energy stress can inhibit ferroptosis via AMPK-mediated ACC phosphorylation and AGEs are capable of downregulating AMPK phosphorylation^12,18^, we hypothesized that AGEs aggravate LPS-induced ferroptosis partly by reducing AMPK activity and downstream ACC phosphorylation.

**Fig. 7:**
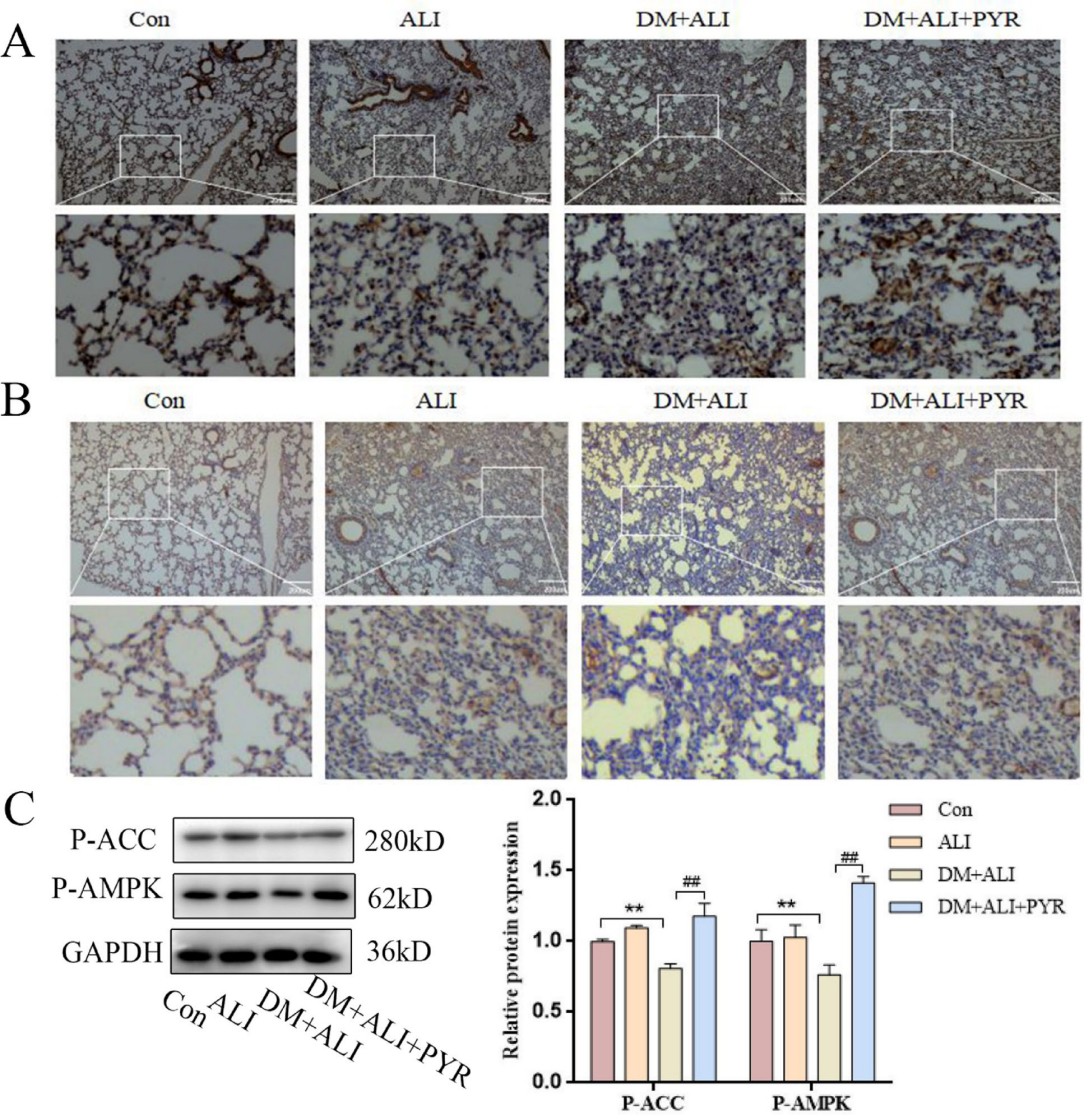
Lowering AGEs levels increases the protein expression of P-AMPK and P-ACC in LPS-induced ALI. (A) Immunohistochemical staining for P-AMPK in lung sections. (B) Immunohistochemical staining for P-ACC in lung sections. (C) Western blot analysis of P-AMPK and P-ACC protein expression in lung tissues. Dark brown staining indicates positive signals. *n* = 5, data are representative of three independent experiments. Scale bars: 200 μm. **P < 0.01, * versus Con group; ##*P* < 0.01, # versus DM + ALI group.

In this study, the protein expression of P-AMPK and P-ACC in lung tissues was detected by immunohistochemistry (Fig. 7A-B) and western blot analysis (Fig. 7C). As shown in Fig. 7C, P-AMPK and P-ACC levels were significantly decreased in the DM + ALI group compared to the Con group, and partially restored in the DM + ALI + PYR group. No significant differences were observed between the Con and ALI groups. We observed similar results in the immunohistochemical outcomes (Fig. 7A-B). These findings indicate that diabetes suppresses AMPK/ACC pathway activity in LPS-induced ALI and that reducing AGEs levels can mitigate this suppression.

**Fig. 8:**
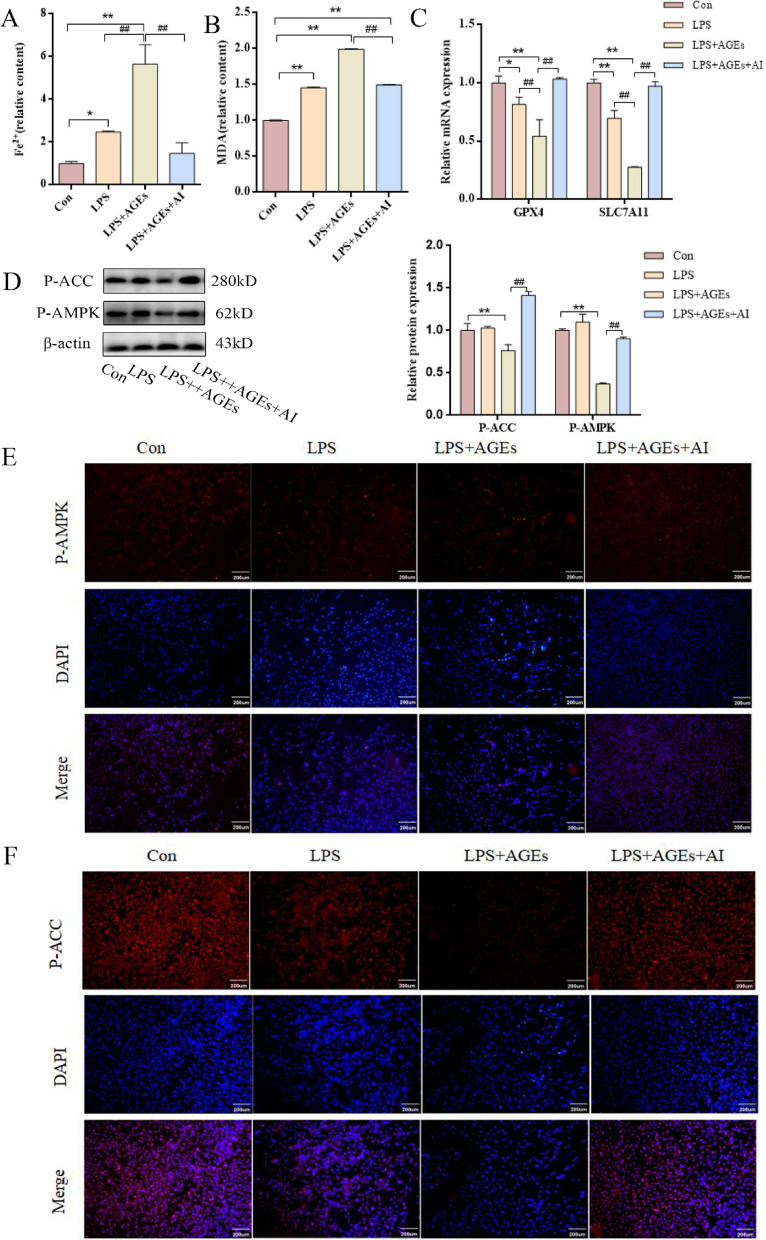
AGEs regulate ferroptosis by modulating ACC phosphorylation via the AMPK pathway. (A) Intracellular Fe²⁺ levels in BEAS-2B cells. (B) MDA levels in BEAS-2B cells. (C) Relative mRNA levels of GPX4 and SLC7A11 in BEAS-2B cells. (D) Protein expression of P-AMPK and P-ACC analyzed by Western blotting. (E) Immunofluorescence staining for P-AMPK in BEAS-2B cells. (F) Immunofluorescence staining for P-ACC in BEAS-2B cells. Data are representative of three independent experiments; scale bars: 200 μm. *P < 0.05, **P < 0.01, * versus Con group; ##*P* < 0.01, # versus LPS + AGEs group.

To further confirm whether AGEs promote ferroptosis via the AMPK/ACC pathway, BEAS-2B cells were co-treated with 5 µg/ml LPS, 100 µg/ml AGEs, and 1 mM AICAR. Ferroptosis biomarkers were subsequently evaluated. As shown in Figs. 8A–C, MDA, and Fe²⁺ levels were significantly reduced, while mRNA expression levels of SLC7A11 and GPX4 were elevated in the LPS + AGEs + AI group compared to the LPS + AGEs group. Western blot analysis revealed that compared to those of the Con group, the protein expression levels of P-AMPK and P-ACC were markedly decreased in the LPS + AGEs group, but significantly restored in the LPS + AGEs + AI group. No significant differences in these protein levels were observed between the Con and LPS groups (Fig. 8D). Immunostaining results were consistent with western blot findings, visually confirming the expression patterns of P-AMPK and P-ACC across the four groups (Fig. 8E-F).

Collectively, these findings suggest that AGEs promote ferroptosis in LPS-induced ALI partly by downregulating AMPK-mediated phosphorylation of ACC. The reversal of this effect by AMPK activation further supports the involvement of the AMPK/ACC pathway in AGEs-mediated ferroptotic regulation.

## Discussion

DM is one of the most prevalent and serious chronic diseases worldwide^19^. Studies have shown that diabetes increases susceptibility to ALI^20,21^, and significantly elevates ALI-related mortality in clinical settings^22^. However, the mechanism by which DM exacerbates ALI remains incompletely understood. In our study, patients with sepsis-related ALI and concurrent DM exhibited significantly higher levels of inflammatory indicators and lower PaO₂/FiO₂ ratios compared to non-diabetic counterparts. These results indicate that DM exacerbates inflammatory responses in patients with sepsis-related ALI.

Our bioinformatic analyses showed that DEGs in both ALI and DM are significantly enriched in pathways related to the ferroptosis and AGE − RAGE signaling pathway in diabetic complications. AGEs are classical biomarkers of DM, and their synthesis increases markedly under hyperglycemic conditions^23^. AGEs play an important role in ferroptosis: AGEs induce cardiomyocyte ferroptosis through the AMPK/NRF2 pathway in diabetic cardiomyopathy^12^, and they enhance ferroptosis in hippocampal neurons via inhibition of the Nrf2/GPX4 axis in diabetic encephalopathy^14^. Moreover, AGEs promote ROS production, which activates the NLRP3 inflammasome and elevates IL-1β levels^24^. Inflammatory signaling can drive ferroptosis^25^, while inhibition of ferroptosis can alleviate inflammation^26^. These findings collectively suggest that AGEs, inflammation, and ferroptosis are interconnected and engage in mutual regulatory crosstalk.

LPS-induced ALI is a well-established experimental model of drug-associated ALI^27^. AGEs have been shown to interact directly with the LPS-HMGB1, forming an LPS-HMGB1-AGEs complex that synergistically amplifies proinflammatory effects^28^. Furthermore, AGEs prolong macrophage responsiveness to LPS, leading to elevations of inflammatory cytokines^29^. In co-culture models, osteoblasts treated with both AGEs and LPS exhibited enhanced expression of inflammatory mediators via multiple signaling pathways, contributing to the pathogenesis of diabetes-associated periodontitis^30–32^. Similarly, osteocytes co-cultured with AGEs and LPS to mimic diabetic periodontitis conditions in vitro underwent ferroptosis, characterized by downregulation of GPX4 and SLC7A11^33^. However, whether AGEs enhance ferroptosis in LPS-induced ALI has not been previously investigated. In this study, we demonstrated that diabetes exacerbates lung injury and increases the recruitment of inflammatory cells and proinflammatory cytokines in the BALF of LPS-induced ALI mice, an effect associated with elevated AGEs levels. Moreover, AGEs are associated with increased markers of ferroptosis in vivo in LPS-induced ALI mice. These findings were further confirmed in vitro, as BEAS-2B cells co-treated with LPS and AGEs exhibited enhanced ferroptotic responses.

We further investigated the underlying mechanism by which AGEs induce ferroptosis in LPS- induced ALI. Bioinformatic analysis of gene expression in ALI and DM demonstrated enrichment in biological pathways related to the regulation of plasma lipoprotein particle levels. GPX4, a crucial regulator that protects against lipid peroxidation during ferroptosis, was notably decreased in the ALI group and further downregulated in the DM + ALI group. These observations led us to hypothesize that AGEs may promote ferroptosis by disrupting lipid metabolism and enhancing lipid peroxidation.

AMPK is a heterotrimeric complex that serves as a critical cellular energy sensor. Upon activation through phosphorylation, AMPK works to restore energy balance and is regulated by a variety of metabolic cues^34^. The level of APMK phosphorylation is modulated during ferroptosis^12,18^. ACC, a downstream target of AMPK, is a key enzyme involved in both fatty acid synthesis and oxidation. ACC catalyzes the conversion of acetyl-CoA to malonyl-CoA, a metabolite essential for de novo lipogenesis and a known allosteric inhibitor of mitochondrial fatty acid oxidation^35,36^. AMPK activation leads to phosphorylation of ACC1 at Ser79 and ACC2 at Ser212, thereby inhibiting ACC activity. This inhibition results in decreased malonyl-CoA levels, promoting fatty acid oxidation and reducing lipogenesis^37^.

Iron-dependent lipid peroxidation is a characteristic of ferroptosis. Coenzyme A and polyunsaturated fatty acids (PUFAs) are converted into acyl-CoA derivatives by acyl-CoA synthetase long-chain family member 4 (ACSL4), which are then re-esterified into phosphatidylethanolamines by LPCAT3 (lysophosphatidylcholine acyltransferase 3)^8^. Then, lipoxygenases (LOX) are involved in the process of lipid peroxidation to form lipid peroxides that disrupt cellular membrane integrity and trigger ferroptosis^38^. Phosphorylation-mediated inhibition of ACC reduces malonyl-CoA production, thereby enhancing fatty acid oxidation and suppressing lipid biosynthesis, which collectively limits lipid peroxidation and increases cellular resistance to ferroptosis^39,40^.

**Fig. 9:**
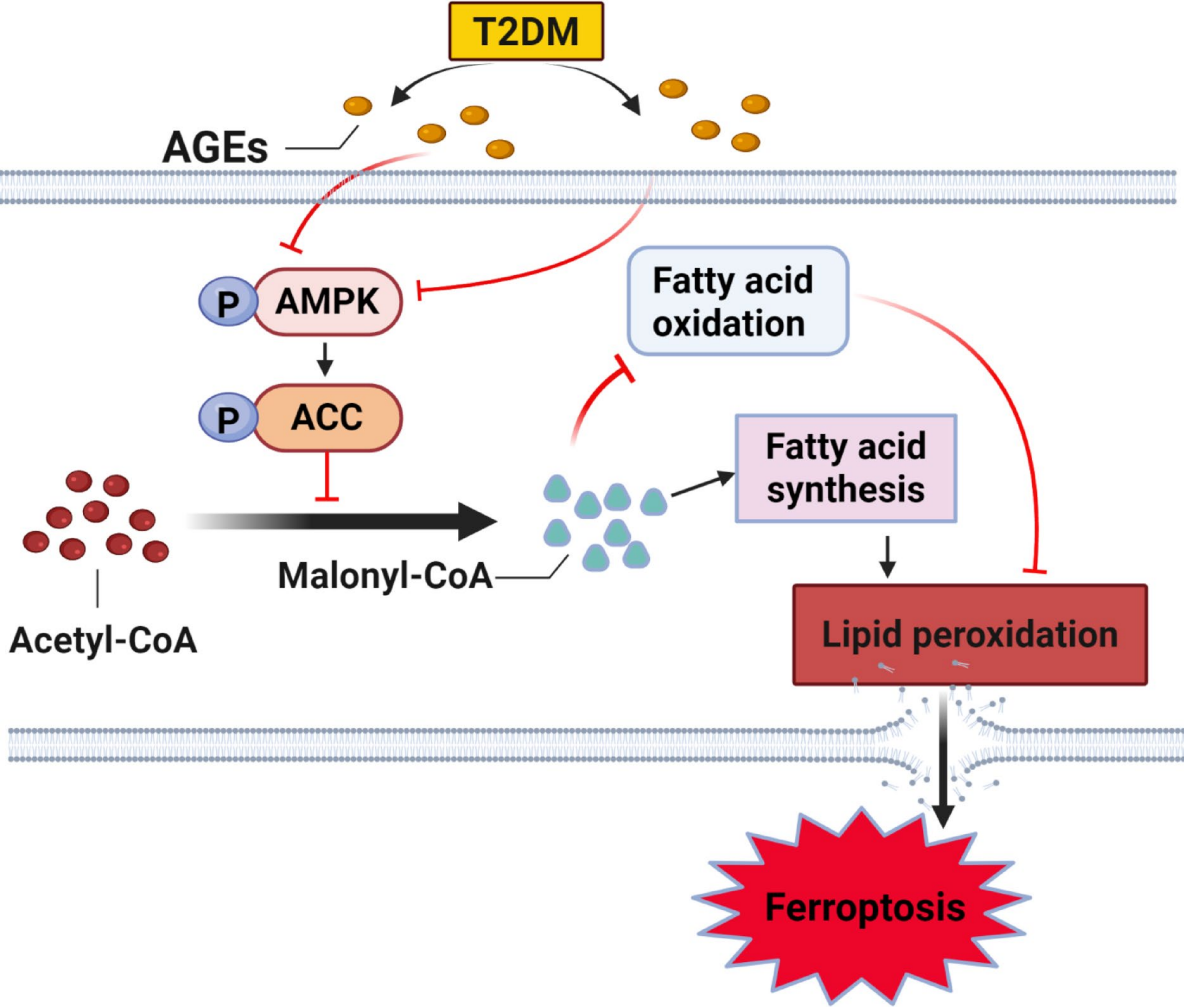
Potential mechanism by which AGEs promote ferroptosis in LPS-induced ALI via the AMPK/ACC pathway. AGEs inhibit the phosphorylation of AMPK and ACC, resulting in increased lipogenesis and reduced fatty acid oxidation. This metabolic imbalance enhances lipid peroxidation, ultimately promoting ferroptosis in alveolar epithelial cells. Black arrows indicate promoting effects; red lines indicate inhibitory effects.

In our study, we observed that AGEs were associated with the exacerbation of ferroptosis both in vivo and in vitro. Furthermore, pharmacological activation of AMPK using AICAR mitigated the AGEs-aggravated ferroptosis, suggesting that AGEs promote ferroptosis, at least in part, through suppression of the AMPK/ACC signaling pathway. The proposed mechanism by which AGEs promote ferroptosis in LPS-induced ALI via the AMPK/ACC pathway is illustrated in Fig. 9.

### Limitations

There are several limitations in our study. First, all findings were obtained from murine models or cell lines, which may not fully reflect the complexity of human ALI pathology. Second, although we observed changes in key ferroptosis markers and signaling molecules, comprehensive lipidomic profiling and direct measurements of lipid peroxidation substrates would strengthen the mechanistic conclusions. Lastly, while we identified changes in AMPK/ACC signaling, it remains unclear whether AGEs act through additional downstream pathways. For example, AGEs markedly reduced p-AMPK expression, which could influence ferroptosis via AMPK/NRF2 signaling or regulate autophagy via AMPK/mTOR signaling^12,41^. The roles of these pathways in AGEs-mediated ferroptosis remain to be determined. Moreover, hyperglycemia activates the STING signaling pathway, which plays a critical role in diabetic complications such as retinopathy and vascular disease^42,43^. STING has also been shown to promote ferroptosis in LPS-induced ALI^44^. However, whether AGEs promote ferroptosis in LPS-induced ALI via the STING pathway remains unknown.

## Conclusions

In this study, our results suggest that diabetes promotes ferroptosis in LPS-induced ALI, with AGEs contributing to this process, at least in part, through the AMPK/ACC pathway. Activating AMPK exerted protective effects against AGEs on ferroptosis in LPS-induced ALI, indicating that modulation of the AMPK/ACC pathway could represent a potential therapeutic strategy for ALI in the context of diabetes.

## Supplementary Information

Below is the link to the electronic supplementary material.

Supplementary Material 1

Supplementary Material 2

## Data Availability

All datasets generated in this study are presented in this article.

## Abbreviations

ALI: Acute lung injury
DM: Diabetes mellitus
AGEs: Advanced glycation end products
LPS: Lipopolysaccharide
DEGs: Differentially expressed genes
BEAS-2B: Bronchial epithelial cells
AMPK: AMP-activated protein kinase
MDA: Malondialdehyde
GPX4: Glutathione peroxidase 4
SLC7A11: Solute carrier family 7 member 11
ACC: Acetyl-CoA carboxylase
ARDS: Acute respiratory distress syndrome
FBG: Fasting blood glucose
CRP: C-reactive protein
PCT: Procalcitonin
WBC: White blood cell
IL-6: Interleukin-6
GEO: Gene Expression Omnibus
KEGG: Kyoto Encyclopedia of Genes and Genomes
GO: Gene Ontology
HFD: High-fat diet
ACSL4: Acyl-CoA synthetase long chain family member 4
LPCAT3: Lysophosphatidylcholine acyltransferase 3
LOX: Lipoxygenases
PUFAs: Polyunsaturated fatty acids
