# Supplementary material for: Advanced glycation end products exacerbate lipopolysaccharide-induced acute lung injury with diabetes by promoting ferroptosis via AMP-activated protein kinase/acetyl-CoA carboxylase signaling
Source: Sci Rep. 2025 Dec 13;15:43755. doi: 10.1038/s41598-025-26647-0 (PMC12705668; doi:10.1038/s41598-025-26647-0)
Supplement: Supplementary file 1 — Supplementary Material 1 [file 41598_2025_26647_MOESM1_ESM.docx]

Table S1. The catalog numbers and the working dilutions of antibodies

| Antibody | Merchant and Catalog Number | Dilution |
| --- | --- | --- |
| GPX4 | Abclonal，A13309 | WB: 1:1000 |
| SLC7A11 | Abclonal，A13685 | WB: 1:1000 |
| P-AMPK | Affinity，AF3423 | IF: 1:100  WB: 1:1000  IHC: 100 |
| P-ACC | Affinity，AF3421 | IF: 1:100  WB: 1:1000  IHC: 100 |
| beta Tubulin Rabbit Monoclonal Antibody | Beyotime，AF1216 | WB: 1:1000 |
| Anti-beta Actin Rabbit pAb | [Servicebio](http://www.baidu.com/link?url=ewhYpW74dkP0wMI6gtO0ATJ1DLiunsbIKGuz8x6HARjg9EkUKXXiQiNhihskNumi)，GB11001 | WB: 1:1000 |
| Anti-GAPDH Rabbit pAb | [Servicebio](http://www.baidu.com/link?url=ewhYpW74dkP0wMI6gtO0ATJ1DLiunsbIKGuz8x6HARjg9EkUKXXiQiNhihskNumi)，GB11002 | WB: 1:1000 |
| HRP-labeled Goat Anti-Rabbit IgG(H+L) | Beyotime，A0208 | WB: 1:1000  IHC: 1:50 |
| Donkey anti-Rabbit IgG (H+L) Highly Cross-Adsorbed Secondary Antibody, Alexa Fluor™ Plus 555 | Invitrogen，A32794 | IF: 1:1000 |

Table S2. Primer sequences (5′to 3′)

| Gene |  | primer |
| --- | --- | --- |
| GPX4 | Forward | 5′- GAGGCAAGACCGAAGTAAACTAC-3′ |
|  | Reverse | 5′- CCGAACTGGTTACACGGGAA-3′ |
| SLC7A11 | Forward | 5′-TCTCCAAAGGAGGTTACCTGC-3′ |
|  | Reverse | 5′-AGACTCCCCTCAGTAAAGTGAC-3′ |
