# Supplementary material for: Advanced glycation end products exacerbate lipopolysaccharide-induced acute lung injury with diabetes by promoting ferroptosis via AMP-activated protein kinase/acetyl-CoA carboxylase signaling
Source: Sci Rep. 2025 Dec 13;15:43755. doi: 10.1038/s41598-025-26647-0 (PMC12705668; doi:10.1038/s41598-025-26647-0)
Supplement: Supplementary file 2 — Supplementary Material 2 [file 41598_2025_26647_MOESM2_ESM.docx]

| 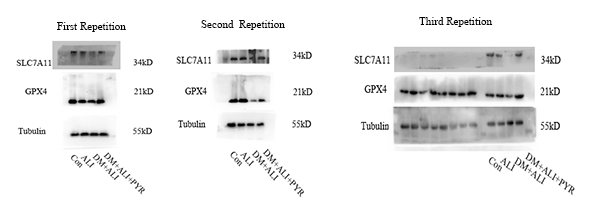 |
| --- |

Figure S1. Western blot images of Fig. 5F. The WB legends of lung tissue of C57BL/6J mice. The images show all blots and replicates of SLC7A11, GPX4, and Tubulin.

| 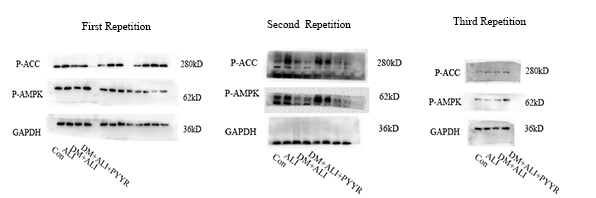 |
| --- |

Figure S2. Western blot images of Fig. 7C. The WB legends of lung tissue of C57BL/6J mice. The images show all blots and replicates of P-AMPK, P-ACC, and GAPDH.

| 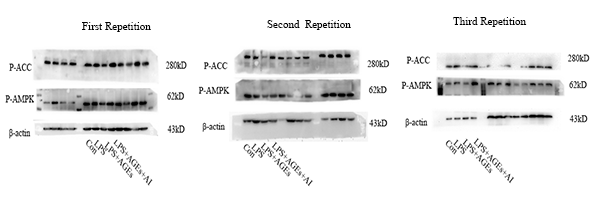 |
| --- |

Figure S3. Western blot images of Fig. 8D. The WB legends of BEAS-2B cells. The images show all blots and replicates of P-AMPK, P-ACC, andβ-actin.
